# Pyomyositis in Nodding Syndrome (NS) patient - a case report

**DOI:** 10.11604/pamj.2013.16.65.2403

**Published:** 2013-10-23

**Authors:** David Lagoro Kitara, Amos Deogratius Mwaka, Henry R Wabinga, Paul Okot Bwangamoi

**Affiliations:** 1 Gulu University, Faculty of Medicine, Department of surgery, P.O. Box 166, Gulu, Uganda; 2 Makerere University, College of Health sciences, Department of Medicine, P.0. B0X 7072, Kampala, Uganda; 3 Gulu University, Faculty of Medicine, P.O. B0X 166, Gulu, Uganda

**Keywords:** Nodding syndrome, Pyomyositis, Uganda

## Abstract

We report a case of Pyomyositis in a 13-year-old boy diagnosed using WHO surveillance definition of Probable Nodding syndrome. Complete blood count showed Leukocytosis with immature granulocytes and atypical lymphocytes. Except for the liver enzymes which were high the renal functions and serum electrolytes were within normal range values. Culture of a pus-swab grew Staphylococcus aureus. Abdominal ultrasound scan showed a focal mass on the internal and external oblique muscles of the right abdominal wall. Incision and drainage was performed. Histology of the muscle showed non-specific inflammation of the external and internal oblique muscles. This finding may highlight some of the other tropical diseases that occur in children with Nodding syndrome.

## Introduction

Nodding syndrome is an unexplained neurologic disorder that has recently been reported among children in several sub-Saharan African countries and primarily among internally displaced persons [1–4]. The primary and characteristic feature is a paroxysmal “spell” in which the head bobs forward repeatedly over a period of minutes; in most cases the children appear unresponsive during the episode [3, 4]. The illness has a clustering of onset between ages of 5 and 15 years [4]. The affected children are stunted, malnourished, dehydrated, mentally retarded and have seizures [4, 5]. We present a 13 year old school boy with NS who had Pyomyositis of internal & external oblique muscles of the anterior abdominal wall and was successfully managed at Gulu Regional Referral Hospital, Uganda.

## Patient and observation

A 13-year-old boy diagnosed with WHO diagnostic criteria of probable NS was referred from Atanga HC III in Pader district where he was enrolled and undergoing care at the nodding syndrome treatment center; he came with a history of progressive swelling and pain in the right lumbar region. The swelling was associated with a high grade fever which was constant and only partially relieved by analgesics. These symptoms were not associated with vomiting, constipation, yellow eyes, loss of appetite or weight loss. The patient reported a history of falling from a tree during one of the nodding episodes in October 2012 and hit his abdomen onto a tree branch. On further probe on his childhood history, his mother reported that he was born normally at home by a Traditional Birth Attendant (TBA) in one of the Internally Displaced peoples (IDP) camps in 2000. She reported that there was an uneventful pregnancy which was carried to term and delivery by Spontaneous vaginal delivery (SVD). She reported that during her pregnancy, she had exclusive feeding on the relief food provided by WFP (beans, yellow posho and cooking oil) during the IDP camps and denies history of ingestion of herbs or medications which caused adverse events during and after the pregnancy. She reported that her child had a normal physical, cognitive and social childhood development before the onset of nodding which began in May 27th 2011 immediately after returning home from IDP camps. The child was enrolled in Atanga treatment centre and was being managed with Carbamazepine, multivitamins and Ivermectin. She reported that in spite of these medications the child continued to have seizures and nodding at least twice a day and had since dropped out of school.

**Figure 1:**
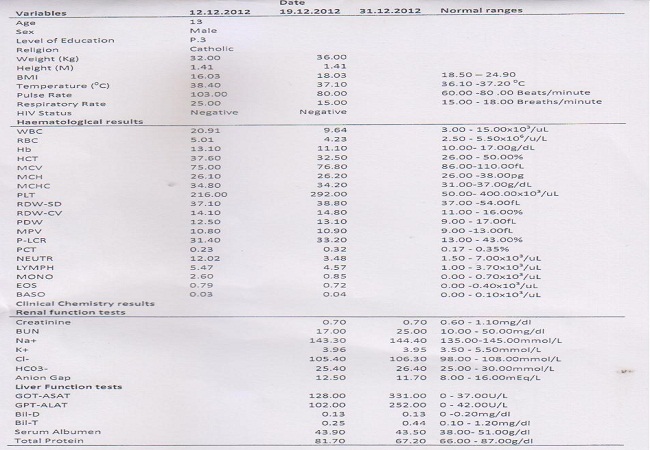
The socio-demographic characteristics and laboratory results of the patient

On general examination, he was dehydrated, febrile and moderately wasted. There was a right lumbar region mass, tender, indurated and non-fluctuant. The spleen and liver were not palpable. There was no renal or supra-pubic tenderness. The rectum was full of faecal material which was of normal colour and consistency. The anal tone was normal and there was no blood on examining fingure. Haematological investigations were conducted and showed neutrophilia, lympocytosis, monocytosis, and eosinophilia. There were immature granulocytes and atypical lymphocytes seen on the peripheral film report. Other laboratory results including liver function tests (ALT, AST) were elevated while serum protein levels were low; renal functions tests (serum creatinine, blood urea and nitrogen level), and serum electrolytes (K+, Na+, Cl-, HC03-) which were within normal ranges (Figure 1). Abdominal Ultrasound showed inflamed internal and external oblique muscles of the anterior abdominal wall.

The patient underwent incision and drainage at Gulu Hospital and wound left open for 14days and thereafter secondary wound closure was conducted. He received supplementary food rehabilitation and his seizure medication was changed to Sodium Valproate 200mg once a day under direct observation therapy (DOTS) and close monitoring of the vital signs. The patient continued to have regular follow up in the surgical ward; seizures and nodding stopped completely from the time of intervention in the hospital. With these interventions for over one month the child had no seizures nor nodding and the child returned to normal life. A subsequent review of the haematological and clinical chemistry findings 2 weeks later showed that renal function tests, serum electrolytes were normal except the liver enzymes level were elevated and were still high (table 1).

## Discussion

The true incidence and prevalence of Nodding Syndrome and Pyomyositis in Uganda are unknown. The socio-demographic characteristic of this child reflected similar findings in those NS children previously investigated [3, 5]. They are generally from poor families and are malnourished [3, 5]. This patient had been receiving medications for symptomatic management of nodding syndrome (anticonvulsants - carbamazepine, multivitamins, Ivermectin, Folic Acid and albendazole) from Atanga health center III for over ten months and was still continuing to have nodding plus seizures at least twice a day. This child developed Pyomyositis of the muscle of the anterior abdominal wall which was successfully managed in the Gulu hospital with a surgical incision and drainage plus antibiotics administration.

Pyomyositis being a suppurative infection of the skeletal muscle has striking clinical features of an inflammatory process [6–9]. This condition is commonly found in tropical regions of East and central Africa, Malaysia, and the pacific islands [6]. Although it occurs in people living in the tropics, it is frequently seen and reported in immunosuppressed patients especially people living with HIV/AIDS in the temperate regions [7, 8].

Pyomyositis has been associated with various conditions such as HIV/AIDS [9–12, 14]; Malnutrition [9, 12]; Diabetes Mellitus[12, 13]; Alcohol abuse [8] and trauma [9, 12]. Of all these risk factors, malnutrition and trauma were the observed risk factors in this patient. The patient was HIV negative and had no reported bleeding or clotting disorders.

### Laboratory investigations

White blood cell counts (WBC): Total white blood cell count was high and the film report did indicate toxic granules to suggest current active infective process. The hemoglobin concentration (Hb) of this patient was within normal range (table 1). Following the surgical procedure, the total WBC returned to normal (table 1).

Renal function tests: The patient had a normal serum creatinine and blood urea nitrogen levels and this indicated that the renal functions were probably normal and was perhaps unlikely that this patient had any intrinsic renal disease. The CDC studies conducted on children with nodding syndrome in Northern Uganda in 2009 also indicated the absence of intrinsic renal diseases [3].

Serum electrolytes: Serum potassium, sodium, chloride and bicarbonate were within normal ranges (table 1).

Liver function tests: The intrinsic liver enzymes AST and ALT were significantly high above the critical clinical threshold and this suggested liver cell injury and may be due to inflammation of the liver. Perhaps some of the anticonvulsant being administered may be a culprit to this but it may perhaps be other unrelated condition to the anticonvulsants.

Anion Gap: This child had a normal anion gap. This means the child was not in a state of acidosis as previously reported in other Nodding children and perhaps that was the reason he had not had any nodding or seizures for the period under our care in Gulu Hospital. He was receiving food supplementation from the time he arrived at the hospital under direct observation from the staff of the institution.

## Conclusion

Children with nodding children are prone to falls and may develop Pyomyositis as result. Health workers caring for these children need to pay particular attention to such children who may have unrelenting fevers and absent malaria parasites in their blood or other evidence to explain the occurrence of the fevers. Children with Nodding Syndrome should continue to receive food rehabilitation concurrently with the symptomatic management under direct observation. Regular check-up of the Liver function tests should be undertaken.
